# Network analysis of human muscle adaptation to aging and contraction

**DOI:** 10.18632/aging.102653

**Published:** 2020-01-07

**Authors:** Craig R.G. Willis, Ryan M. Ames, Colleen S. Deane, Bethan E. Phillips, Catherine L. Boereboom, Haitham Abdulla, Syed S.I. Bukhari, Jonathan N. Lund, John P. Williams, Daniel J. Wilkinson, Kenneth Smith, Fawzi Kadi, Nathaniel J. Szewczyk, Philip J. Atherton, Timothy Etheridge

**Affiliations:** 1 Department of Sport and Health Sciences, College of Life and Environmental Sciences, University of Exeter, Exeter EX1 2LU, UK; 2 Biosciences, University of Exeter, Exeter EX4 4QD, UK; 3 MRC-ARUK Centre for Musculoskeletal aging Research and National Institute of Health Research, Biomedical Research Centre, Royal Derby Hospital Centre, School of Medicine, University of Nottingham, Derby DE22 3DT, UK; 4 Department of Surgery, Postgraduate Entry Medical School, Royal Derby Hospital Centre, School of Medicine, University of Nottingham, Derby DE22 3DT, UK; 5 School of Health Sciences, Örebro University, Örebro 70182, Sweden

**Keywords:** skeletal muscle, aging, contraction, network analysis, candidate target discovery

## Abstract

Resistance exercise (RE) remains a primary approach for minimising aging muscle decline. Understanding muscle adaptation to individual contractile components of RE (eccentric, concentric) might optimise RE-based intervention strategies. Herein, we employed a network-driven pipeline to identify putative molecular drivers of muscle aging and contraction mode responses. RNA-sequencing data was generated from young (21±1 y) and older (70±1 y) human skeletal muscle before and following acute unilateral concentric and contralateral eccentric contractions. Application of weighted gene co-expression network analysis identified 33 distinct gene clusters (‘modules’) with an expression profile regulated by aging, contraction and/or linked to muscle strength. These included two contraction ‘responsive’ modules (related to ‘cell adhesion’ and ‘transcription factor’ processes) that also correlated with the magnitude of post-exercise muscle strength decline. Module searches for ‘hub’ genes and enriched transcription factor binding sites established a refined set of candidate module-regulatory molecules (536 hub genes and 60 transcription factors) as possible contributors to muscle aging and/or contraction responses. Thus, network-driven analysis can identify new molecular candidates of functional relevance to muscle aging and contraction mode adaptations.

## INTRODUCTION

Skeletal muscle represents the most abundant tissue constituent of the human body, accounting for approximately 40% of total body mass in healthy individuals [1]. Many key physiological processes are dependent on skeletal muscle, including locomotion, whole-body substrate metabolism and temperature regulation [2], and its maintenance is thus critical for physical function and health [3]. This is particularly relevant to chronological aging, where the progressive loss of skeletal muscle mass and strength that accompanies advancing age (termed ‘sarcopenia’ [4]) associates with decreased functional capacity [5], metabolic disease [6], reduced quality of life [7] and ultimately increased mortality rates [8]. Given the worldwide aging population is projected to almost double by 2050 [9], promoting healthy skeletal muscle across the lifespan remains a major public health priority.

Resistance exercise (RE) training offers the most effective lifestyle intervention for enhancing muscle mass and strength in youth [10] and older age [11]. Nonetheless, older muscle displays blunted hypertrophic and functional gains following chronic RE training [12, 13], the molecular mechanisms of which are incompletely defined (e.g. [14, 15]). Traditional RE involves repeated episodes of lengthening (eccentric, ECC) and shortening (concentric, CON) contractions, which can each be distinguished by their distinct mechanical (ECC) and metabolic (CON) characteristics [16]. It is thus plausible that poorer adaptation of aging muscle to RE may be due at least in part to unique molecular and/or functional responses to individual contraction modes. Consistent with this we have recently demonstrated, via classical differential expression analysis, age-related and mode-dependent transcriptional responses of muscle to contraction [17]. Notably, although both young and older muscle showed large overlap of CON *vs.* ECC transcriptional changes, older muscle exhibited: (i) a CON-specific downregulation of mitochondrial genes and upregulation of blood vessel development- and cell adhesion-related genes, and; (ii) an ECC-specific response without clear ontological functional relevance [17], perhaps reflecting some mechanically-mediated stochasticity [18].

Whilst these findings provide insight on the transcriptional basis of muscle adaptation to aging and contraction mode, muscle is a complex organ comprised of highly coordinated and diverse molecular systems that cannot be surmised by changes in expression of single molecules. Additionally, although reductionist approaches highlight that individual genes/ subsets of genes can be central to muscle regulation (e.g. highly connected ‘hub’ genes and transcription factors governing classes of genes), key molecular drivers of adaptation do not necessarily display evidence of differential regulation in isolation [19]. As such, standard differential gene-level analyses overlook such biological complexity, and meaningful information captured by a transcriptomic experiment can remain hidden [20]. Moreover, the (usually large) lists of differentially expressed genes remain difficult to prioritise downstream, due to the relationships between statistical significance, fold change and biological significance often being discordant [20]. Thus, although the utility of traditional differential gene expression analyses is invaluable, such approaches often lead to a drowning in information but starvation of knowledge [21].

Co-expression network analysis is an alternative approach for encompassing the complexity of entire molecular systems whilst probing putative individual molecules that govern, for example, muscle adaptation to age and exercise. Such an approach accounts for the intrinsic organisation of the transcriptome by placing focus on the co-regulation of genes as a function of expression similarity [22]. Groups of genes displaying a tightly coordinated expression pattern can then be further analysed using established network-centric methods to sequentially deduce the pathways and key molecular drivers modulating a given phenotypic response. Accordingly, co-expression network analysis represents a biologically-motivated data reduction scheme that can provide novel understanding of complex biological phenomena beyond that attained via standard differential gene-level analysis alone [21, 23]. Indeed, recent meta-analyses highlight the potential utility of network analyses for understanding human aging [24]. However, its application to individual tissues, and particularly muscle, is limited. In the present work, we thus establish a co-expression network analysis pipeline for advanced data-driven insight into novel molecules regulating human muscle adaptation to aging and individual contraction modes. Additionally, we elucidate functionally relevant molecular networks by establishing their association to end-point physiological measures of muscle strength.

## RESULTS

### RNA-sequencing dataset

The current work utilised our RNA-sequencing dataset originally presented in [17], containing whole-transcriptome gene expression data generated from the skeletal muscle (*m. vastus lateralis*) of young (18-30 y) and older (65-75 y) individuals at baseline (BL) as well as 5 h following isolated unilateral CON and contralateral ECC leg extension exercise. After appropriate processing of the raw RNA-sequencing data (see ‘methods’), normalised expression values for 12044 genes across 36 samples (6 young BL; 6 young post-ECC; 5 young post-CON; 7 older BL; 5 older post-ECC; 7 older post-CON) were obtained for downstream analyses.

### Gene co-expression network generation

**Figure 1:**
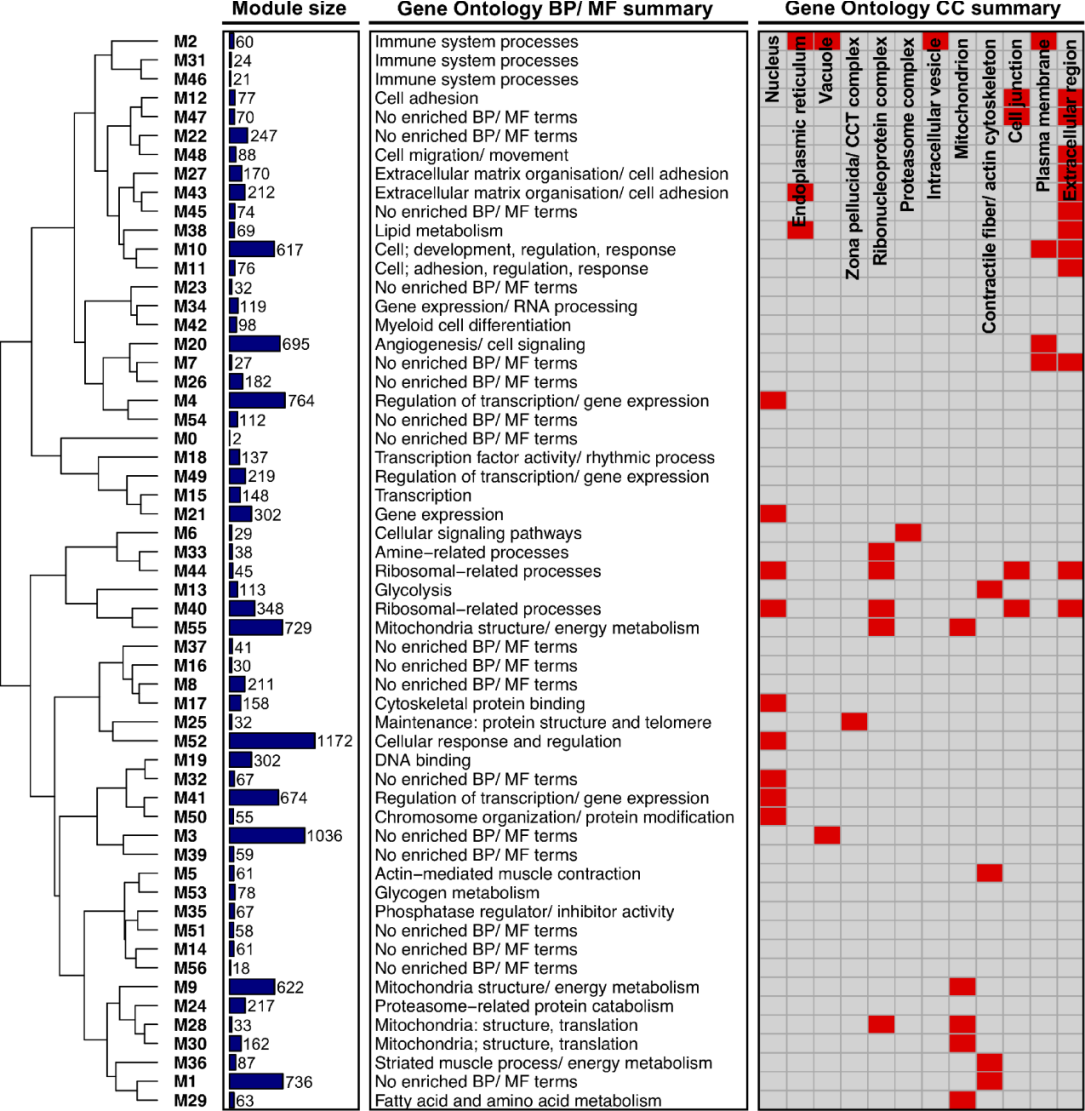
**Higher-order functional organisation of the co-expression network.** Network modules (labelled ‘M*i*’) are hierarchically clustered based on their eigengene correlations (using average linkage and ‘1 – correlation’ as a distance metric). Modules closer together in the dendrogram therefore have a more similar expression profile. Also given is the size of each module (depicted as a bar chart), a summary of each module’s enriched Gene Ontology (GO) Biological Process (BP)/ Molecular Function (MF) terms, and a summary of each module’s GO Cellular Component (CC) terms (provided as a heatmap, where red shading denotes that a module is enriched with GO terms related to a given CC).

As an initial step in our network-driven pipeline, we modelled interactions among genes in our dataset by constructing a gene co-expression network using the underlying methods of weighted gene co-expression network analysis (WGCNA) [25]. In particular, a signed gene-wise network was assembled in order to sustain a greater distinction between gene ‘activation’ and ‘repression’ [21]. Application of signed-WGCNA subsequently returned an approximately scale-free gene co-expression network comprising 56 distinct groups of genes (i.e. network ‘modules’, labelled M1 - M56), based on the similarity of their expression pattern changes across all samples. Modules ranged in size from 18 genes (M56) to 1172 genes (M52) with the mean and median module sizes being 215 and 88 genes, respectively. The expression profiles of genes within a given module were condensed into a single representative profile of module gene expression, defined as the module eigengene (calculated as the 1^st^ principle component of module gene expression). The module eigengene were then utilised for subsequent downstream analyses at the module level (where appropriate; see ‘methods’). Notably, only two genes were unable to be non-trivially clustered into a particular module and were thus assigned to the module ‘M0’. Gene Ontology (GO) enrichment analysis of module gene sets together with hierarchical clustering of module eigengene (based on their correlation) highlight that, overall, the network portrays a logically organised set of modules which are diverse in aspects fundamental to the innate maintenance/ function of skeletal muscle (Figure 1). Lists of all genes comprising each module can be viewed in Supplementary Table 1, with all enriched GO terms for each network module provided in Supplementary Table 2.

### Molecular networks, ‘hub’ genes and transcriptional regulators of older muscle

**Figure 2:**
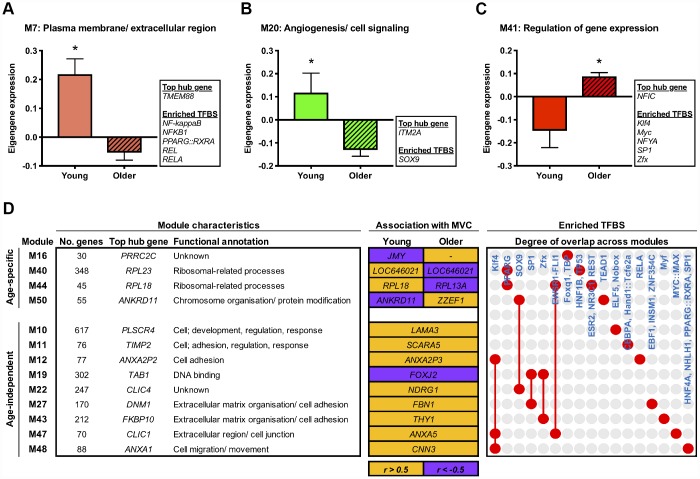
**Age-related molecular networks and candidate molecules in resting muscle.** Panels (**A**–**C**) Network modules displaying a divergent co-expression pattern between ages at baseline. Box inserts show the top ranked hub gene, and all identified enriched transcription factor binding sites (TFBS) for each module. Data are mean ± SEM. *FDR < 5%. Panel (**D**) Network modules that significantly associate (*P* < 0.05) with baseline maximal voluntary isometric contraction (MVC) in either an age-dependent or age-independent manner. Orange shading denotes a positive relationship and purple indicates a negative relationship. Also shown is each module’s top ranked hub gene, the hub gene ranked highest among the module genes by gene significance to MVC at baseline (i.e. within the upper quartile of module genes ranked by their gene significance to baseline MVC (shown in orange/ purple shaded boxes)), and enriched TFBS. Red dots/ connecting red lines indicate whether a given TFBS is enriched in the genes of one or more MVC-related module.

To explore molecular changes that might underpin muscle adaptation to aging *per se*, we subsequently established gene modules with composite expression altered by age in the basal state. Accordingly, we applied differential analyses to the module eigengene and identified three network modules with an aging-dependent expression profile (FDR < 5%). Two of these modules represent molecular networks downregulated in older muscle, comprising genes enriched for plasma membrane/ ECM (M7) and angiogenesis/ cell signalling (M20) GO terms (Figure 2A and 2B). The third age-related module (M41) represents a molecular network upregulated with aging, containing genes involved in the regulation of gene expression/ transcription (Figure 2C). A complete list of differentially regulated network modules is given in Supplementary Table 3.

Next in our analysis pipeline we sought to identify genes that might represent key molecular candidates of muscle aging. We therefore applied two further biologically-motivated data reduction techniques to each age-related molecular network, namely: (i) hub gene identification, by filtering module genes for those of highest intramodular connectivity [26], and; (ii) transcriptional regulator prediction, by analysing module genes for enriched transcription factor binding sites (TFBS) [27]. In doing so, we deduce a vastly refined set (*vs.* 1396 genes across all age-related modules) of 95 putative molecules (84 hub genes and 11 transcriptional regulators) that may be key drivers of aging-induced muscle dysregulation. For example, among the ~6% of genes in M20 (containing 695 genes) identified as modular hubs were a number of caveolin and G protein-related genes whilst SOX9, a transcription factor important for musculoskeletal development and angiogenesis, was the sole predicted transcriptional regulator of such a pathway (Figure 2B). Full lists of hub genes and predicted transcriptional regulators for each pertinent module are given in Supplementary Table 4.

### Molecular networks associated with basal muscle function

To determine possible molecular networks of functional relevance in the context of human age, we established network modules whose expression profile (i.e. eigengene) correlated to muscle strength (maximal voluntary isometric contraction, MVC) at baseline in either an age-dependent or age-independent manner. Notably, we found four modules displaying age-dependent association with muscle strength at baseline (age-eigengene interaction, *P* < 0.05), such that their relationship with MVC in older muscle was the direct converse of that in younger muscle (Figure 2D). These included two ribosomal-related pathways (M40, M44) related positively to baseline MVC in younger muscle but negatively in older muscle. A further nine modules were found to associate with basal muscle strength irrespective of age (*P* < 0.05; partial correlation analyses with age as a covariate) (Figure 2D). These were mainly modules positively correlated with baseline MVC and enriched for cell adhesion- and extracellular matrix (ECM)-related GO terms (M11, M12, M27, M43, M47, M48).

Our hub gene and predictive transcription factor analyses were then used to identify key age-(in)dependent molecular drivers of basal muscle strength. Interestingly, the two ribosomal-related molecular networks showing age-dependent association with basal muscle strength (i.e. M40, M44) also show some commonality in enriched TFBS, namely for *PPARG*. Moreover, several hub genes identified within these two networks hold a shared relevance to mechanistic target of rapamycin (mTOR) signalling (*RPL41, RPS13*, *RPS21*, *RPS29* (M40), *RPL13A* and *RPL18* (M44)) [28] and too display strong evidence of an age-dependent link to basal muscle strength (based on their ‘gene significance’ (GS) to basal muscle strength; see ‘methods’). Some common regulatory themes also appear among modules showing age-independent association with baseline muscle strength. For example, within each of the six cell adhesion-/ ECM-related modules that positively correlate with basal muscle strength irrespective of age, the hub gene ranked highest by its GS to basal MVC (independently of age) collectively form a set featuring several prominent membrane-associated genes, that is; *ANXA2P3* (M12), *ANXA5* (M47), *CNN3* (M48), *FBN1* (M27), *SCARA5* (M11), *THY1* (M43). Three of these modules (M12, M47, M48) also contain genes under the predicted control of KLF4, a zinc-finger transcription factor important for cell-cell binding. Other cell adhesion-related modules were similarly enriched with TFBS for zinc-finger transcription factors (SP1 (M27), ZFX (M43)), as was module M19 (SP1 and ZFX) – the single module negatively correlated with basal MVC independent of age, containing genes involved in ‘DNA binding’ (Figure 2D). Complete lists of module-strength correlations can be found in Supplementary Table 3, with corresponding GS scores for hub genes provided in Supplementary Table 4.

### Contraction mode-related molecular networks in young and older muscle

**Figure 3:**
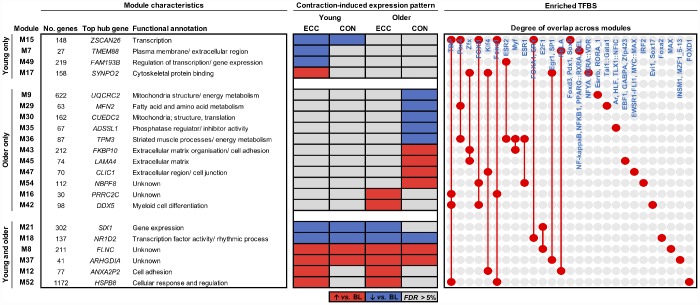
**Molecular networks and candidate molecules of the aging muscle contractile response.** Modules shown are those with a co-expression profile responsive to ECC and/or CON contraction in young adults, older adults or both. Red and blue shading denote significant post-exercise upregulation and downregulation relative to baseline (BL), respectively (FDR < 5%). Also provided is each such module’s top ranked hub gene, and their enriched transcription factor binding sites (TFBS). Red dots/ connecting red lines indicate whether a TFBS is enriched in the genes of one or more contraction-induced module.

We next applied our network-driven analysis pipeline to determine candidate molecular signatures of young and older muscle acutely (5 h) after isolated CON or isolated ECC exercise. Differential analyses of the module eigengene identified a total of twenty-one modules with an expression profile ‘responsive’ to contraction (i.e. altered *vs.* baseline) in an age- and/or contraction mode-(in)dependent manner (FDR < 5%; Figure 3). Two modules also displayed age-specific suppression post-CON when comparing absolute post-exercise expression patterns (M9 and M55, both related to mitochondrial biogenesis/ metabolism) (Supplementary Table 3).

Irrespective of contraction mode, younger muscle alone displayed downregulation of a molecular network enriched for ‘transcription’ GO terms (M15), containing genes under the predicted control of several forkhead box transcription factors (FOXI1, FOXQ1, FOXA1, FOXD3). Whilst no CON-unique network modules were found in younger muscle, ECC contraction distinctly associated with the upregulation of a ‘cytoskeletal protein binding’ pathway (M17) and the downregulation of molecular pathways related to the plasma membrane/ ECM (M7) and the regulation of transcription (M49). Thus, downregulation of gene pathways involved in controlling transcription occurred in younger muscle after both CON and ECC (M15) and ECC alone (M49). Nonetheless, the predicated transcriptional regulators of these pathways were entirely distinct, perhaps indicating separate molecular regulation of gene expression in younger muscle by isolated ECC contraction *vs.* contraction *per se* (i.e. irrespective of contraction mode) (Figure 3).

Older muscle also presented several ECC- and CON-specific network module expression changes, that were not found in younger muscle (Figure 3). For example, ECC contraction upregulated a myeloid cell differentiation-related molecular network (M42), whose associated hub genes include an RNA helicase (*DDX5*, top hub gene) and several nuclear pore complex interacting protein family members (*NPIPB3*, *NPIPB4*, *NPIPB5*). Interestingly, older muscle displayed a post-CON upregulation of several ECM-related modules (M43, M45, M47) and downregulation of a number of mitochondrial-/ energy metabolism-related modules (M9, M29, M30, M36), two of which appear under the putative control by the PAX4 transcription factor (M29, M36). Additionally, the hub genes of module M36 were almost exclusively sarcomeric structure genes (i.e. myosin light/ heavy chain, troponin and tropomyosin genes).

In addition to the above, several network modules were identified to represent pathways of age-independent contractile regulation (M8, M12, M18, M21, M37, M52) (Figure 3). Among these included two network modules with an expression profile upregulated uniquely by ECC contraction in both young and older muscle, enriched with genes involved in cell adhesion (M12) and cellular regulation (M52). A further two such modules also display age-independent upregulation, but instead do so irrespectively of contraction mode (i.e. increased post-CON and post-ECC) (M8, M37). Whilst the gene sets of these two particular modules show no ontological functional enrichment, the top hub gene of each (*FLNC* (M8), a sarcomeric Z-disc protein involved in striated muscle (dys)function and; *ARHGDIA* (M37), a Rho-GTPase inhibitor) putatively serves to function in cytoskeletal organisation/ remodelling.

### Molecular networks associated with acute post-exercise functional responses

**Figure 4:**
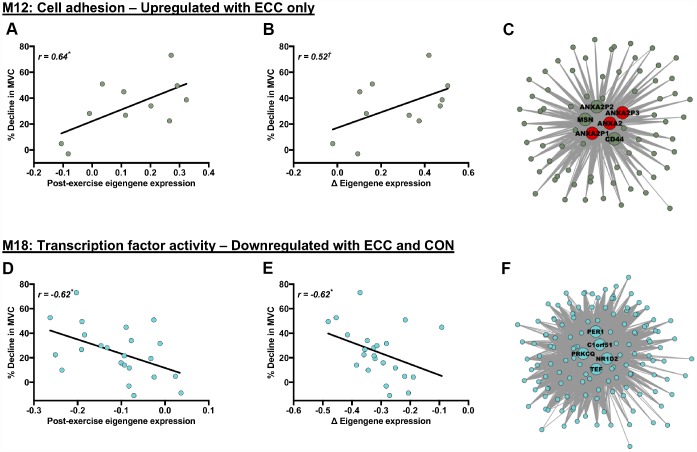
**Molecular networks and candidate molecules related to the acute post-exercise muscle functional response.** Panels (**A**, **B**, **D** and **E**) Scatterplots showing relationships between post-exercise declines in MVC (% decline from baseline) and contraction-induced eigengene expression patterns (for exact post-exercise eigengene expression values (**A**, **D**) and changes (Δ) in eigengene expression from baseline to post-exercise (**B**, **E**)). Panels (**C**) and (**F**): corresponding module visualisations for M12 (Panel **C**) and M18 (Panel **F**). Of note, larger, annotated nodes in panels (**C**) and (**F**) highlight module hub genes: red shading depicts individual hub genes highly linked to the % decline in MVC (i.e. within the upper quartile of module genes ranked by their gene significance to the post-exercise decline in MVC). Module visualisations were generated using Cytoscape (v3.5.1) [56]. *P < 0.05 and ^†^*P* < 0.1 with |r| > 0.5 in all cases, using Pearson’s or Repeated Measures correlation where appropriate.

Both acute ECC and CON contractions induced variable declines in MVC 5 h post-exercise, in both young and older individuals. We therefore investigated potential relevance of contraction-regulated modules (as shown in Figure 3) to the acute post-exercise functional response, by correlating each of their specific post-exercise eigengene patterns with the corresponding 5 h post-exercise muscle strength responses (% MVC decline from baseline) (e.g. we correlate post-ECC eigengene patterns with ECC-induced strength declines across age for a module upregulated by ECC *per se*, etc.). Whilst none of the young- or older-specific, contraction-regulated modules had a post-exercise eigengene pattern that correlated with the respective contraction-induced MVC decline, significant correlation were found for two of the network modules (M12 and M18) regulated by contraction irrespectively of age (Figure 4). M12 is a cell adhesion-related module, which is upregulated by ECC contraction and has a post-ECC eigengene pattern (absolute and delta change) that positively associates with ECC-induced MVC declines (Figure 4A and 4B). M18 is a ‘transcription factor activity’-related module, which is downregulated by both CON and ECC contraction and has a post-contraction eigengene response (absolute and delta change) that negatively associates with contraction-induced strength declines (i.e. pooled ECC- and CON-induced MVC decrements) (Figure 4D and 4E). We then explored whether any hub genes within these two modules (6 for M12, 5 for M18) might also be highly relevant to the acute post-exercise functional response of muscle. None of the hub genes within module M18 fell among the highest ranked module genes based on their individual association (i.e. GS) with acute contraction-induced (post-ECC and -CON) declines in muscle strength (Figure 4E). However, for module M12, 3 of the 6 hub genes were among the top module genes when ranked by their individual association with ECC-induced strength declines and were exclusively Annexin A2 genes (Figure 4C), with known functions in regulating muscle repair.

## DISCUSSION

Establishing molecular causes of and countermeasures to poor skeletal muscle aging remains an important goal to ensure optimal human health and performance across the life course. Resistance training currently offers the most effective lifestyle countermeasure to mitigate age-related muscle loss and dysfunction, yet associated muscle mass and strength gains remain blunted in older *vs.* younger individuals [12, 13]. To gain new insights into potential molecular drivers of muscle aging and contraction responses, we employed an advanced network-driven pipeline through which we: (i) define molecular networks regulated by aging and/or contraction; (ii) establish primary candidate targets of age- and contraction mode-(in)dependent muscle adaptation, and; (iii) predict molecular networks and molecular targets of potential functional relevance to human age and acute contraction responses.

### Network analysis for identifying molecular signatures of muscle adaptation

In our previous work we utilised traditional differential expression analysis to examine the impact of age and contraction mode on muscle transcriptomic responses to RE [17]. Here, we extend the insight gained from transcriptomic datasets by establishing an analysis pipeline that defines molecular interaction networks regulated by aging and/or contraction via WGCNA; an advanced co-expression network tool for integrating gene-level data into a higher-order, systems-level framework [29]. We identify several gene patterns that are consistent with those observed through our traditional differential expression analysis [17], for example the CON-specific suppression of mitochondrial genes and upregulation of cell adhesion-related genes in older muscle. Unlike our standard differential gene-level analysis, however, network analysis was also able to provide ontological insight for ECC-specific signatures. Notably, older muscle alone showed a post-ECC upregulation of a myeloid cell differentiation pathway. Since immune cells of the myeloid lineage have a significant role in directly (i.e. acting on muscle) and indirectly (via angiogenesis and fibrosis regulation) enhancing muscle regeneration [30], this network might represent an interesting molecular feature unique to aging muscle that ensures recovery of post-ECC muscle damage is comparable to that of younger adults [31]. Our findings thus corroborate the increased power of network-based analysis for detecting new, biologically-relevant transcriptional signatures of skeletal muscle beyond that possible from standard differential gene-level analysis alone [23].

### Unravelling potential molecular drivers of muscle adaptation to aging and exercise

A major advantage of network analysis is the ability to systematically reduce an entire transcriptome to a handful of predicted molecular regulators of physiological adaptation [22]. On the premise that key mechanistic candidates likely include centrally located ‘hub’ genes [26] and/or transcription factors strongly enriched for regulatory binding sites in a given set of co-expressed genes [27], we established a refined list of 536 molecular hubs from the 8135 genes across all age-/ contraction-regulated and strength-related modules, along with an even smaller complementary set of putative transcriptional regulators (60 in total) to these molecular networks. Whilst individual discussion of all identified hub genes and predicted transcription factors is beyond the scope herein, this provides an experimentally tractable list of putative molecular targets for further hypothesis generation. For example, SOX9 is identified as a predicted transcriptional regulator of genes comprising the angiogenesis network signature downregulated in older muscle *per se.* SOX9, a purported modulator of tissue angiogenesis [32, 33], might thus represent an interesting candidate molecule influencing age-related impairments in muscle angiogenesis [34], which is itself implemented in the aetiology of aging muscle decline [35]. Hub gene analysis further highlights a possible role of abnormal ribosomal processing in muscle aging – ribosome-related networks that positively associate with basal MVC in the young but negatively in the old contain several hub genes with relevance to mTOR signalling [28] that similarly show strong age-discordant association to basal muscle strength. Thus, corroborating recent pathway analysis of aging muscle alone [36], deregulated ribosomal and protein synthetic machinery appears a prominent molecular feature of aging muscle weakness. Additionally, supporting our previous report of mitochondrial gene insensitivity to CON exercise in older muscle [17], network analysis confirms and extends this to identify PAX4 as a common transcription factor predicted to regulate multiple mitochondria-/ energy metabolism-related networks suppressed post-CON in older muscle. Because PAX4 is implicated in mitochondrial biogenesis and function [37] and mediates second stage muscle atrophy in mice [38], it may present a promising target for future aging and exercise studies. We therefore establish network analysis as a powerful data reduction scheme for generating new, biologically meaningful insight into the molecular drivers of muscle adaptation to age and contraction mode.

### Molecular networks that associate with post-exercise functional adaptation

Large variability in individual responsiveness to physical activity has emerged as a fundamental principle of exercise physiology [28, 39]. Identifying molecular networks displaying expression changes that scale with the magnitude of post-exercise strength responses might provide likely candidates for explaining this inter-individual variability and present strong putative regulators of post-exercise functional responses. Of our twenty-one network modules responsive to ECC and/or CON, we observed two that significantly correlated with corresponding post-exercise functional changes. Several cell adhesion- and ECM-related molecular networks positively associated with baseline muscle strength in both young and older muscle. High habitual expression of cell adhesions might therefore promote and/or be a consequence of the highest levels of basal muscle strength. Additionally, one such cell adhesion module was responsive to ECC *per se* and positively correlated with the extent of ECC-induced strength declines. Individuals displaying minimal post-ECC expression of cell adhesion genes thus appear pre-disposed for resilience against ECC-induced loss of muscle strength. Since the gene that encodes the rapidly acting sarcolemmal and muscle repair-mediating protein Annexin A2 [40] was identified as a recurrent molecular hub within this network module, Annexin A2 might represent a viable target for understanding cell adhesion-mediated muscle damage and repair. Lastly, a general ‘transcription factor activity’-related module downregulated irrespective of contraction mode also negatively correlated with strength declines imposed by contraction *per se* (i.e. pooled post-ECC and -CON strength decrements). Whilst this functional annotation is too broad to be informative, the most highly connected modular hub, NR1D2, influences muscle lipid homeostasis and hypertrophic capacity via strong regulation of interleukin-6 and myostatin, respectively [41]. This network module and its associated hub genes may, therefore, provide insight into the early signals of muscle responsiveness to exercise *per se*.

In summary, we present predictive network-driven analysis as a powerful addition to traditional differential expression transcriptomic analyses. Although limited sample size implores some caution when inferring wider biological relevance, WGCNA performs strongly for network construction and hub gene identification when applied to both smaller (~20 samples) [42], paired design [43] datasets, including within exercise physiology using comparable sample sizes [44]. Nevertheless, extrapolating true aging/ exercise effects requires a much larger sample size than that presented herein, and future studies verifying these gene signatures are warranted. Thus, whilst further validation is needed (e.g. larger sample sizes and quantitative/mechanistic analysis of identified molecules), our data reduction pipeline is effective in identifying an experimentally tractable and biologically plausible set of molecular candidates driving muscle adaptation in the context of human age and the contraction response, including many that appear functionally relevant. The current work therefore holds immediate potential to accelerate the discovery process of primary regulators of age-related muscle decline and exercise responsiveness. Our findings can thus expedite mechanistic understanding of aging-exercise interactions, and help develop optimal exercise interventions to counteract sarcopenia and associated health concerns.

## MATERIALS AND METHODS

### Overview of experimental procedures

The experimental procedures are in line with those outlined in detail previously [17]. In brief, eight young (mean ± SEM: age, 21 ± 1 y; body mass index, 23 ± 2 kg.m^-2^; 80% ECC 1 repetition-maximum (1-RM), 211 ± 14 kg; 80% CON 1-RM, 122 ± 11 kg) and eight older (age, 70 ± 1 y; body mass index, 26 ± 1 kg.m^-2^; 80% ECC 1-RM, 155 ± 1 kg; 80% CON 1-RM, 79 ± 6 kg) healthy, exercise-naïve (i.e. no history of partaking in regular, structured exercise within the previous year) males volunteered for this study. Participants completed 7 sets of 10 unilateral CON contractions and 7 sets of 10 contralateral ECC contractions of the knee extensor muscle group at 80% of their CON and ECC 1-RM’s, respectively. Muscle biopsies were collected from the *m. vastus lateralis* of a randomised leg under local anaesthesia (1% Lidocaine) at baseline (BL, -96 h; serving as reference to both contraction conditions), and then from the *m. vastus lateralis* of each leg at 5 h following the termination of its corresponding exercise bout (i.e. post-ECC and post-CON). Muscle tissue was snap frozen in liquid nitrogen and stored at −80°C until analysis. Muscle strength was measured as a marker of muscle function and was quantified by assessing maximal voluntary isometric contraction (MVC) of the quadriceps of each leg (Humac Norm, CSMI, Stoughton, USA), both before and 5 h post-exercise. All experimental procedures were approved by the University of Nottingham Faculty of Medicine and Health Sciences Research Ethics Committee and conformed to the Declaration of Helsinki. Informed consent was obtained from all subjects prior to their participation.

### Generation of RNA-sequencing data

The current work makes use of the raw RNA sequencing data reported in [17], which can be found within the NCBI BioProject database (https://www. ncbi.nlm.nih.gov/bioproject/) under the SRA accession PRJNA509121, and that were generated as previously described [17]. In short, total RNA was extracted from frozen muscle tissue using TRIzol reagent, and samples with a sufficient RNA integrity (RIN ≥ 5.7; 39 samples in total) sequenced using the Illumina HiSeq 3000/ HiSeq 4000 platforms (Beijing Genomics Institute). All raw reads were of sufficient quality (established using FastQC; Babraham Bioinformatics) and were thus subsequently aligned to the human genome (hg38) using Bowtie2 [45], with further processing of alignment files undertaken via SAMtools [46]. Reads mapping to known exons were then counted in an un-stranded manner using featureCounts [47] and with the human genome annotation as a reference (hg38).

### RNA-sequencing data processing

Genes displaying consistently low expression across samples (read count < 10 in at least 80% of samples) were first removed and the read counts for retained genes then normalised to reads per kilo-base per million mapped reads (RPKM). RPKM values were further log transformed (Log2[RPKM + 1]), before outlier samples were identified and removed using the inter-sample correlation (ISC) metric [48]. In particular, samples with a mean ISC < 2.5 SD below the mean ISC for the entire dataset were excluded. A total of 3 outlier samples (1 young BL; 1 young post-CON; 1 older post-ECC) were identified and removed. Log2[RPKM + 1] expression for 12044 genes across 36 samples were subsequently obtained for downstream analyses.

### Gene co-expression network construction

A signed gene co-expression network was constructed from the processed expression dataset using the weighted gene co-expression network analysis (WGCNA) methods implemented in the WGCNA package for R [49]. Briefly, an adjacency matrix (*Adj*) quantifying the connection strength between each pair of genes in the dataset was derived as *Adj* = |0.5 x (1 + *Corr*)| *^ß^*, where *Corr* is the matrix of Pearson’s correlation coefficients that indicate the degree of similarity in expression pattern between any two given genes across the samples. The exponent *ß* ensures greater disparity between strong and weak connections and is chosen with the intent of attaining an approximately scale-free network [25]. An appropriate value of *ß* can be chosen using the scale-free topology fitting index metric (signed-R^2^) [25], and on this basis, a value of *ß* = 17 was chosen to achieve a signed-R^2^ value ≥ 0.85. The adjacency matrix was then converted into a topological overlap matrix (TOM), in which each entry provides a measure of the relative inter-connectedness (‘common connections’) between a given pair of genes. A dissimilarity topological overlap matrix was subsequently calculated as ‘1-TOM’ and used to obtain a network tree through hierarchical clustering using average linkage as a distance metric.

### Identification of network modules

Network modules were determined from the corresponding network tree using an adaptive and iterative branch cutting scheme (*cutreeDynamic* algorithm) [50], with a medium sensitivity (*deepSplit* = 2) and moderate minimum module size (*minClusterSize* = 15) considered when identifying modules. Module gene expression profiles were summarised by their ‘eigengene’ (1^st^ principle component of module expression), and modules with similar expression profiles (Pearson’s correlation coefficient between their eigengene ≥ 0.9) subsequently merged. All modules were accordingly assigned a numerical label for identification, with the module labelled as ‘M0’ containing un-clustered genes. The module labelled ‘M0’ was therefore not included in any downstream analyses beyond module functional annotation (see below).

### Functional annotation of network modules

Functional annotations of network modules were derived on the basis of their gene compositions by undertaking enrichment analysis of Gene Ontology (GO) terms. Analysis was performed using the online Database for Annotation, Visualisation and Integrated Discovery (DAVID, version 6.8) [51], with each of the three GO categories (‘Biological Process’, BP; ‘Cellular Component’, CC; ‘Molecular Function’, MF) considered. The corresponding background gene list used consisted of all genes comprising the network. Of note, analyses were limited only to network genes with an attributable Entrez ID uniquely recognised within the DAVID database (11733 genes). Enrichment was calculated using a modified Fisher exact test and GO terms in each category with a Benjamini-Hochberg (BH) [52] corrected *P* < 0.05 were accepted as being enriched.

### Determining modular expression differences with age and/or contraction

The effects of age and contraction on module expression patterns were established by undertaking differential analysis of module eigengenes using the LIMMA package for R [53]. In brief, a linear mixed effects model was fitted to the eigengene of each module, with a group means parameterisation of experimental condition (all possible age-sample point permutations) included as a fixed effect. A random effect of subject was also included to account for the correlation between samples from the same participant. An empirical Bayes method was then applied to calculate moderated *t*-statistics through shrinkage of estimated sample variances towards a pooled estimate [54], and pairwise comparisons subsequently made between sample points within each age group as well as between ages at each sample point. Statistical significance was accepted for a global BH corrected *P* < 0.05.

### Establishing modular links with muscle functional parameters

Potential links between module expression patterns and muscle function were elucidated to by assessing relationships between the module eigengene and MVC values, which were considered in the basal state (i.e. at baseline) and in response to exercise (where appropriate). That is, relationships between baseline eigengene and baseline MVC values (average of both legs) were quantified for all network modules either separately for each age group (using Pearson’s correlation) or for age groups together (using partial correlation with age as a co-variate), dependent on whether the baseline eigengene-MVC association for a given module appeared to be influenced by age (significant (*P* < 0.05) interaction between age and baseline eigengene expression in the corresponding linear regression model). For post-exercise analyses, relationships between post-exercise eigengene patterns (both exact post-exercise values and changes from baseline) and post-exercise changes in MVC (% decline from baseline) were determined for those modules with a contraction-induced expression profile, in their respective contraction-regulated contexts (e.g. young ECC-specific etc.), using Pearson’s correlation or Repeated Measures correlation [55], as appropriate. Statistical significance was accepted in all instances for which |*r*| > 0.5 and *P* < 0.05.

### Hub gene assessment of pertinent network modules

Key molecular drivers within age-, contraction- and/or muscle strength-related modules (i.e. modular ‘hub genes’) were defined on the basis of their scaled intra-modular connectivity (‘relative intra-connectedness’) [26]. Specifically, the within-module connectivity for each gene from a given module was calculated by summing its connection strengths to all other genes from the same module, and subsequently divided by the maximum within-module connectivity value for that module to attain a scaled intra-modular connectivity measure. Genes with a scaled intra-modular connectivity value ≥ 0.7 were considered hub genes. The hub genes of functionally-significant network modules were also assessed on the basis of their gene significance (GS) to muscle function, which was quantified by the absolute correlation coefficient of the relationship between individual gene expression and MVC, as determined in similar fashion to the eigengene-MVC associations outlined above. In this regard, hub genes of these particular modules were further prioritised by those falling within the upper quartile of the given module’s comprising genes when ranked by their GS.

### Uncovering putative transcriptional regulators of pertinent network modules

Putative transcriptional regulators of age-, contraction- and/or muscle strength-related network modules were identified by checking for enriched transcription factor binding sites (TFBS) in their comprising genes using oPOSSUM-3 Single Site Analysis [27]. The corresponding background list comprised all genes used to construct the network. Of note, large modules (> 500 genes) were represented by their upper third most connected genes, and all analyses was limited only to network genes with an attributable Ensembl ID recognised within the oPOSSUM-3 database (11187 genes). All JASPAR CORE vertebrae profiles with a minimum specificity of 8 bits were queried during analyses, and putative TFBS pertaining to a conservation cut-off of 0.4 and similarity matrix score threshold of 85% were examined for enrichment in the 5 kb upstream/ downstream region encompassing transcription start sites. For a given module, TFBS with a corresponding Z-score (rate of occurrence in module *vs.* network) *and* Fisher score (proportion of hits in module *vs.* network) ≳ the mean + 1.5 SD of their respective distributions were considered as enriched.

## Supplementary Material

Supplementary Table 1

Supplementary Table 2

Supplementary Table 3

Supplementary Table 4
